# Medical Legislation at Albany

**Published:** 1898-04

**Authors:** 


### MEDICAL LEGISLATION AT ALBANY.

DURING the session of the New York legislature for 1898 a multiplicity of medical bills has been offered, though few possess abiding interest to the medical profession. These bills, for the most part, are of a minor character and appear to stand very little chance of becoming laws. There are three measures of importance, however, and of which we wish to speak in particular.

The most dangerous bill introduced up to the present writing is entitled, An act regulating and legalising the practice of osteopathy in the state of New York and fixing penalties for the violations of the provisions of this act. Of all the ridiculous pretensions to "science" there is only one fake that outranks osteopathy and that is so-called christian science. The one thing to be said in favor of the former over the latter is that it really does something—namely, it rubs and manipulates; whereas the latter appeals wholly to the supernatural and pins its faith on the mysterious. It is a strange commentary on the intelligence of the state of New York to find it seriously considering the legalisation of osteopathy, while Kentucky and other states in the south and west are turning it down as unworthy of attention. It, at best, is a form of massage which it would be as ridiculous to legalise and dignify into a system of medical practice as it would be to so honor the office of an ordinary bath attendant.

The next bill that merits attention is the so-called dispensary bill introduced by a faction of the medical society of the county of New York. The sponsors of this bill came up to Albany to attend the last meeting of the medical society of the state of New York, with the avowed intention of forcing the society to indorse and father the dispensary bill. They were so cocksure of carrying their point that they had previously instructed their delegates "to present the dispensary bill to the society for indorsement; to cast their votes in favor of such indorsement and to vote only for those candidates for officers of the state society who individually pledge themselves to indorse the passage of the bill by the legislature, and to vote against any and all candidates for office who may be opposed to said bill or to any similar bill or legislation." Upon reaching Albany the friends of the dispensary bill soon realised that they had overdone the matter in their hidebound, ironclad instructions, hence the bill was not presented for the consideration of the state medical society. While it cannot be denied that dispensary abuses exist, this bill does not provide the proper way to correct them. It is about as absurd to commit dispensaries to the supervision of the state board of charities as it would be to place trained nurses under similar supervision. We shall expect to see the latter quickly follow in case the former is accomplished.

The third bill to demand adverse criticism is one presented by the medical society of the county of Montgomery to regulate the practice of midwifery. We deem it unwise for the legislature to pass bills of this nature. If this becomes a law each county in the state, one by one, will be encouraged to ask for similar privileges until there will be created sixty separate midwifery boards each with a standard of its own. County midwifery boards as established have not been conspicuous successes. The state should commit this subject to a single board or commission which should be empowered to regulate and standardise the practice throughout the commonwealth.

One of the liveliest hearings of the session took place in the senate chamber, Thursday, March 16, 1898, on Senator Coggeshall's bill amending the public health act in relation to the unlawful practice of medicine, making it a misdemeanor for a person not a physician to "charge or receive any fee or pecuniary reward for a service rendered by himself or an assistant employed by him or under his control in the treatment of or prescription for any disease, defect or deformity, except in giving the treatment known as massage."

We are in ignorance as to the origin of this bill. It certainly was not introduced at the instigation of the medical society of the state of New York, hence ought not to have been given standing or the dignity of a hearing. However, it furnished an opportunity for one of the most amusing, if not silly, scenes ever witnessed at the Capitol.

At 2.30 o'clock Senator Brush, chairman of the senate public health committee, opened the hearing in the senate chamber. The floor and the woman's gallery were packed with christian scientists. Women predominated in the representation and their zeal in opposing the bill was manifest as speaker after speaker defended their faith.

Senator Coggeshall sat with the committee and quietly listened to the arguments and maledictions launched at his bill. When the last speaker for the opposition had finished, he arose and smiled at the women. In a speech glowing with compliments and flowery metaphor, he regretted that the draft of the bill should have been inadvertently so broad as to include the christian scientists. He expressed sorrow that he had caused so many good women to journey so far and asked the committee to amend the bill so as to except christian scientists from its operation.

His announcement was greeted with a cheer and a wild waving of handkerchiefs and umbrellas. Hundreds of women made a rush for the bar of the senate and surrounded the tall senator from Oneida. They struggled with each other to shake hands with him and when Senator Brush insisted that the floor should be cleared in order that the business of the committee might proceed, the excited women fairly bore Senator Coggeshall from the chamber to the senate parlors, where they made him the center of an impromptu reception which lasted nearly an hour.

The introduction of such bills, unauthorised by an incorporated medical society, is a damage to the cause of advanced medical education and state control. It will be erroneously supposed by many that this bill was introduced by the regular profession of medicine, whereas it was in reality merely political claptrap, unworthy of the dignity of the legislature of the Empire state.

The current session, happily, has been free, thus far, from any general assault upon the medical practice act, which we trust is an indication that such efforts henceforth will cease. This will leave the officials charged with the execution of the law free to turn their attention to the improvement of its working details.

---

THE New York *Medical Record* prints the following in its issue of March 12, 1898:

> In the recent editorial on osteopathy, the credit for prosecuting quacks should have been given to the medical society of the county of New York and not the state medical society. The latter never does anything in that line unless perhaps in an obstructive way.

We are surprised that so good a journal as the *Record* should make such a *faux pas* as it has in the foregoing paragraph. Every physician familiar with the workings of medical societies knows that the duties referred to by the *Record* pertain to the county societies and that the state medical society has naught, whatever, to do with apprehending or prosecuting violators of the medical practice law. Moreover, the state society was instrumental in framing and obtaining the passage of the act and it borders on the absurd to charge it with being obstructive in relation to prosecutions under the statute.

